# “They can smell the ‘short-termness’ on you”. Student learning and sense of belonging in the Arctic

**DOI:** 10.1080/17482631.2026.2739684

**Published:** 2026-09-26

**Authors:** Rannveig Grøm Sæle, Kristin Benjaminsen Borch, Iris Borch

**Affiliations:** a Department of Psychology, Faculty of Health Sciences, UiT The Arctic University of Norway, Tromsø, Norway; b Department of Community Medicine, Faculty of Health Sciences, UiT The Arctic University of Norway, Tromsø, Norway; c Centre for Faculty Development, Faculty of Health Sciences, UiT The Arctic University of Norway, Tromsø, Norway

**Keywords:** Sense of belonging, well-being, mental health, higher education, professional roles, mental health services

## Abstract

**Background:**

Student well-being is recognized as central to academic success, yet little is known about how extreme, isolated learning environments may shape this relationship.

**Purpose:**

To explore student's and staff's perception of student well-being and learning at the University Centre in Svalbard (UNIS), focusing on the Arctic context.

**Methods:**

We used a qualitative, exploratory design, conducting semi structured interviews and focus groups with students and staff at UNIS.

**Results:**

We identified four themes: Arctic isolation, nature as the learning environment, belonging and identity, and extended roles. Participants emphasized strong field‑based pedagogy, high motivation, and close student–staff bonds that foster learning and belonging. Simultaneously, geographic isolation, seasonal variability, logistical constraints , and limited local mental‑health services amplifies challenges. Staff frequently adopt extended caregiving roles without formal training, blurring boundaries and increasing workload. Participants expressed a need for on‑site psychological services, targeted staff training, clearer role definitions, and a focus on mental health promotion.

**Conclusion:**

UNIS has engaged staff and motivated students, and the environment foster valuable learning opportunities. Simultaneously, there are some important psychosocial vulnerabilities. A whole‑university approach combining on-site support services, staff development, and embedded mental health promotion would better safeguard student well-being and sustain learning outcomes.

## Introduction

Student well-being is essential for academic success, and most higher education institutions have in place services for psychological support and learning guidance. However, there are institutions situated in remote areas where such services are not available. One example is the University Centre in Svalbard (UNIS), located in Longyearbyen, one of the world's northernmost permanent settlements. The Norwegian island of Spitsbergen—in Norway known as Svalbard—is located at 78° North, with a one and a half-hour flight to Tromsø, the nearest mainland airport offering regular departures. Students at UNIS come from all over the world and typically stay for periods ranging from six weeks to half a year, some even longer. Many are drawn to UNIS because of the distinctive setting—close to nature, with rapidly changing weather and light conditions, including the midnight sun, polar night, and northern lights. The presence of polar bears adds both fascination and a sense of danger, requiring students to be trained in handling firearms if they go outside of the village. Studying at Svalbard offers a unique experience. However, the fluctuating student population, harsh climate and living conditions, strict restrictions on movement, and the small, isolated community may also contribute to mental health difficulties and a demanding learning environment.

While student mental health and belonging have been studied extensively before, little is known about how these constructs unfold in small, geographically extreme institutions where adequate formal psychological support is unavailable. This gap is important to address, not only because the conditions may intensify known challenges, but also because they may produce qualitatively different consequences than in other remote institutions. UNIS serves as a critical case with an extreme context in which tensions become especially apparent, but examining UNIS is relevant beyond its local interest, offering an amplified view of dynamics that may be less visible in conventional university environments. Hence, we developed a project where we conducted interviews with both students and academic and administrative staff to explore their experiences regarding learning environment, mental health support, daily student life, and integration into the Longyearbyen community.

Mental health problems are increasingly prevalent among students (Knapstad et al., 2021; Sheldon et al., 2021; Storrie et al., 2010). Good mental well-being at university, on the other hand, is associated with academic success, like improved concentration, problem-solving, and exam performance (Hughes et al., 2022). These associations between learning and well-being implicate that institutions must act on both, making sure to have adequate services for students with mental health struggles (Hughes & Spanner, 2024; Hughes et al., 2025). Psychosocial outcomes as sense of belonging, social support and academic and social self-efficacy predict both grades and persistence (Richardson et al., 2012; Swanson et al., 2021). Important aspects of student sense of belonging are extracurricular activities and connection with others (Ménard et al., 2024), together with the surroundings of the institution (Ahn & Davis, 2020). Surroundings involve both geographical location, environmental and cultural context, and the physical learning environment. Such factors may influence to what extent students feel they belong to the place where the institution is located—their temporary home. In the unique nature and cultural environment at Svalbard, one could expect this element to be quite strong. At the same time, most students are present at UNIS for shorter periods. It is therefore interesting to investigate how they feel welcomed, and are able to participate, in the society.

The University Centre in Svalbard (UNIS) is the northernmost higher education institution in the world. Established in 1993, UNIS operates as a state-owned limited company under the Norwegian Ministry of Education and Research (The University Centre in Svalbard, 2019). UNIS has about 30 academic staff living in Longyearbyen, plus almost 200 guest lecturers and adjunct positions. About 700 students from 40 countries enrol each year; about half of whom come from Norwegian institutions. UNIS does not offer full degree programs, but a range of courses at Bachelor's, Master's and PhD levels. The courses include field work and/or cruises and have a specific and dedicated arctic focus within five disciplines (biology, geology, geophysics, technology and safety). The student groups in each course are in general small (i.e., 10–20 students), and dropout rates are low. Previously, UNIS hosted two centres of excellence in teaching, bioCEED and iEarth, with a strong focus on student learning environment and inclusion.

Longyearbyen is an old coal mining settlement, with a population of approximately 2400 people. The last operating coal mine was closed in 2025, and today, tourism and research have increasingly become important sources of employment. Polar night is present from end of October to middle of February, while the midnight sun shines from mid-late April to mid-late August. There are approximately 300 polar bears living permanently at Svalbard, making it mandatory to always bring a gun when traveling outside the settlement of Longyearbyen. People from all countries that have signed the Svalbard Treaty (Svalbardtraktaten, 1920) can freely settle in Longyearbyen, and it is a largely international community. The underlying condition for living there is to have a source of income. Longyearbyen is not intended to be a community where people spend their whole lifetime, and health- and welfare facilities are limited. The town has a small, local hospital, but no regular mental health service. If further services are needed, Norwegian residents must contact their home municipalities on the mainland, while foreign nationals must turn to their respective home countries. During the recent years, the Norwegian government has made efforts to ensure that the local government keep its Norwegian connection, by introducing a requirement for foreign citizens to have lived at least three years on mainland Norway to be eligible to vote in the local elections (Royal Decree No. 100, 2022).

The limited access to mental health services, combined with challenging weather and living conditions, makes the situation vulnerable for students. In addition, since only single courses are offered, the student population is constantly changing according to the duration of the courses they attend. Our aim was therefore to systematically explore the nature of the challenges the students face.

In this paper, we build the analysis of the empirical data on principles from a whole university approach developed by Hughes and Spanner (2024). The whole university approach is a framework anchored in the WHO Ottawa Charter and comprises four domains. The first domain, **Learn**, covers a range of factors broadly related to learning, including onboarding of first-year students into university, pedagogy, curriculum design, inclusivity, assessment strategies, support for learning, and transitions through, and out of, the study program. The framework acknowledge that staff need both adequate competence and clear, appropriate boundaries, to support students in a sustainable way. Domain two, **Support**, includes support services, risks (for instance related to mental health crises or suicide), external partnerships and pathways, and information sharing. Universities should have systems and competence in place to identify students at risk and to intervene, via available internal or external services. The third domain is **Work**, and it deals with staff well-being and development, including equipping managers and staff with necessary competence to support students' well-being and be able to respond to students' mental health needs, while also maintaining healthy boundaries. Finally, the domain **Live** is about promoting good mental health to students and staff through creating supportive environments and safe communities, and to make sure that students are becoming socially integrated and that they feel that they belong to the student community.

We judge this approach to be well-designed for our investigations into the UNIS students for two key reasons: (1) UNIS, as a rather small institution, already embodies many characteristics of a whole university approach, and (2) elements from all the different domains of the framework are highly relevant for our research question.

Our research question is as follows: How do students, teachers and administrative staff at UNIS perceive student's well-being and learning in this specific educational setting, and how does the psychosocial, physical, organizational, and pedagogical learning environment support or hinder both? Findings from this study can inform how remote and resource-limited institutions may develop training and staff support, with relevance beyond UNIS to other isolated or high-risk settings, for instance field stations, offshore programs or expedition-based courses.

## Methods

The study has a qualitative, exploratory design, with semi-structured interviews and focus groups.

### Inclusion criteria and descriptions of the sample and context

All students and staff enrolled or employed at UNIS in 2025 met the inclusion criteria and were eligible for participation. We recruited participants actively over two waves when we were ourselves present at UNIS; once in April and once in November, representing different seasons where some students were present when the dark times turn to light, and others during the gradually colder and darker autumn semester. All participants who responded to our invitation, were included in the study.

In both groups, we interviewed Norwegian and foreign participants, hence the sample was reflecting the international environment at UNIS. The individual interviews lasted approximately 60 min and were conducted by one interviewer, while the focus group sessions lasted approximately 90 min, and involved one interviewer and one observer, as recommended in focus group literature (Krueger et al., 2015). During the focus groups, two of the authors were present; one leading the discussions, another supporting the facilitator and encouraging participation from all attendees, ensured that the conversation remained on topic. All authors participated in some of the interviews. By combining the individual interviews and focus group interviews and the perspectives from students and staff, we aimed to achieve a more comprehensive understanding of the phenomena learning and well-being at UNIS.

**Student participants.** Student were invited to participate through flyers posted at the UNIS campus, announcements sent via e-mail lists maintained by personnel at UNIS, and via social media channels managed by student representatives. Students signed up via QR codes for available time slots and provided consent through an online form (Nettskjema; University of Oslo). Consenting participants also responded to a short questionnaire with background information.

We conducted four individual interviews (due to scheduling issues) and two focus interview (three participants in each). Three of the individual interviews were conducted online, the rest of the student interviews were conducted in-person at UNIS. In total 10 students participated, six males and four females, aged 21 to 29 years. They studied geology, biology, geophysics, technology, and safety, representing all five study fields at UNIS. Five were bachelor's students and five master's students.

The discussions were guided by a semi-structured interview guide, which included open-ended questions aiming to explore students' experiences and opinions on the topic of interest. Participants in this group are referred to as students.

**Academic staff participants.** Employed academic staff at UNIS were invited to participate in the study through strategic recruitment, i.e., we approached staff that had been suggested by UNIS colleagues. Seven participants consented to individual interviews, three male and four female, representing biology (3), geology (2), geophysics (1) and safety (1). Four of the consenting teachers had been involved in the centres of excellence in teaching, and their responses may therefore not be representative for the general academic staff. Each interview lasted between 60 and 90 min, four were conducted in-person, three online. We used a semi-structured interview guide with open-ended questions aiming to explore teachers' experiences and reflections. In this paper they are referred to as teachers.

**Administrative staff participants.** Employed administrative staff at UNIS were invited to participate in the study. Three study advisers accepted and participated in an online focus group interview that lasted for 90 min. Participants are referred to as administrative staff.

When we refer to both academic and administrative participants, we use staff.

### Ethical considerations

The focus group discussions and individual interviews were audio-recorded through Nettskjema, a secure survey solution for researchers developed and hosted by University of Oslo (nettskjema@usit.uio.no) and transcribed verbatim using Autotekst/OpenAI Whisper V3 on the same secure server. Prior to the start of each focus group and individual interviews, participants were informed both written and orally about the study's purpose, the voluntary nature of their participation, and their right to withdraw at any time without penalty. Confidentiality and anonymity were assured by using pseudonyms during the transcription process. In addition, given the small size of the UNIS community, we refrain from providing detailed sample characteristics, to protect participants' privacy. In-person interviews took place at UNIS, in offices or meeting rooms, while online interviews were conducted via the author's university's version of MS Teams, approved for confidential data. Informed consent was obtained from all participants. The project is approved by Sikt, the Norwegian Agency for Shared Services in Education and Research (reference: 118414).

### Analysis

We conducted a qualitative thematic analysis of the interview data inspired by Braun and Clarke (2021). NVivo 15.4 (Lumivero, 2026) was used to structure the interview data during the analysis. All three authors read all interviews transcriptions and participated in the analysis process. Initially we pilot-coded one interview together in NVivo Cloud, a collaborative function in NVivo that allowed us to work in the same file. We discussed the raw data and created descriptive codes relevant to the research questions. In the next stage, each researcher coded a selection of interviews independently based upon the codes we had agreed on. Further, we met and identified categories describing more latent meaning. During this stage of the iterative analysis, the Whole university framework described above, provided the authors insights and informed the categorization of the emerging themes. Thus, the framework guided our discussions about what the empirical data was telling us. The discussions about the data in light of the framework, ensured a more shared understanding of the findings and strengthening the trustworthiness of the analysis (Creswell & Poth, 2018).

We met several times during the analysis, moved back and forth between the coded and categorized data, and got new insights by discussing and renaming the categories. New and deeper understandings of what the data was really telling us was adjusted throughout the process of analysis and discussions within the researcher team. The authors' professional background as both health professionals and educational researchers may have influenced the analyses and interpretation, as well as the choice of theoretical framework. We aimed to be self-reflective about our background and discussed how it may have influenced the research process and particularly the analysis. One of us is trained as a clinical psychologist, two are physiotherapists. Our research backgrounds are within learning sciences, student learning environment, faculty development, university pedagogy, epidemiology and health promotion, which might bias our interpretation, but also provides relevant competence for the study. We were transparent about our background and roles to the students and informed them that we met them as independent educational researchers with no employment or commitment to UNIS in the interview. In addition, collaborations with The Norwegian Arctic Student Welfare Organization had already made us aware of some of the challenges at UNIS, thus influencing how we designed the interview guide.

We initially identified five themes 1) Arctic Isolation, 2) Nature as the Learning Environment, 3) Flexibility and Adaptability, 4) Belonging and Identity, and 5) Extended Roles, but we eventually merged theme 2 and theme 3 because of overlapping content. The themes are used as headlines when we present the results below.

## Results

### Arctic isolation

Participants emphasized that one of the most distinctive aspects of being at UNIS and Longyearbyen, compared to other institutions and student cities, is the isolation. Students find themselves far away from the mainland and many of the facilities they normally have access to. At Svalbard, they are accompanied by only a relatively small group of people. Additionally, they must contend with specific physical conditions and dangers, especially related to polar bears.

*“There are quite extreme contrasts to many of the local contexts where people come from, especially for foreign students with the long polar night and the security situations, the threats that exist both related to animals and also the climate and the Arctic when you are out in the field, or (…).” (Student 4)*

*“But in the first couple of weeks, they're terrified that everything in Svalbard is trying to kill them because that's what they've been shown in the safety videos, you know, all the ways that you can get into trouble up here. So I think that can be intimidating.” (Teacher 2)*

The students attend a mandatory safety course in their first week, including rifle training, but not all manage to get the necessary permits to carry a rifle in time. In those cases, they will have to ally up with other students with access to rifles to be able to go outside of town. This restricts their independence in their free time, but also in the field, and can limit their learning opportunities.

*"Because then everyone had to go out and do their own fieldwork, but she didn't have weapons, and she was depended on doing, bringing someone who had access to weapons, and do her fieldwork. And then it also took place during evenings." (Student 3)*

The environment changes dramatically with the seasons, which affects the opportunities for exploration. In the summer and early fall, before the snow comes, it is more difficult to get around, and some students describe themselves as being trapped. They can attend the typical tourist activities, but these would often be expensive.

*"You feel a little trapped in this time [during summer]. You are just staying in this city and the building. There is a road 10 kilometres one way and 10 kilometres back. If you don't have a boat, you won't really get anywhere." (Student 4)*

Another aspect of isolation is how it can amplify problems students already struggle with. The teachers are aware of the effect the isolation and the environmental conditions can have on their students, especially in combination with the lack of available help. One teacher told us about a student considering coming to UNIS, who had also revealed that they suffered from chronic depression.

*“And so, yeah, my colleague basically told them that, okay, yeah, if I'm completely honest, maybe this is not the best place for you because we do not have these resources here. And we have conditions that can really amplify depression.” (Teacher 4)*

Being part of a small group of students is mainly described as positive. However, it also creates some challenges. Unlike in bigger student cities, you can't easily avoid people you don't get along with. Students are surrounded by the same individuals every day and can't really be anonymous.

*“I guess it's quite vulnerable since it's such a close, small community” (Teacher 2)*

One teacher connected this to a pressure to live up to certain expectations of how life should be and what one should experience while being at Svalbard:

*“the fact that the people around you uh you cannot choose and you can't get away from that's uh if there's someone you really don't like uh in Oslo university you don't bump into them too often here you will see them every day uh the smallness of the society and somehow this expectation of what your life should be on Svalbard. So everyone will feel bad because they don't have the chance to do big ski trips or… There's always someone who comes up with this photo of a polar bear drying its fur in front of a glacier during a sunset. That's just how it is. And seeing those for five months without experiencing it, being so close but so far, that is slow poison. So I think that's the biggest thing.” (Teacher 6)*

The description “slow poison” may suggest that social comparison, limited anonymity, and unmet expectations work together in creating a possible feeling of inadequacy or exclusion. It points to how a potential idealization of Svalbard life can become a subtle source of pressure.

### Nature as the learning environment

The courses offered at UNIS are characterized by a strong emphasis on field-based learning, with the natural environment as the primary learning arena. Across the different disciplines, including geology, biology, geophysics, and technology, the learning output is to enable students to develop competence in observing, documenting, and collecting empirical data under Arctic conditions.

*“There's no reason for somebody to come here and then sit in a classroom and just hear lectures. They can do that in Oslo.” (Teacher 1)*

Consequently, the physical learning environment is predominantly outdoors, which provides unique educational benefits.

*“And now they're just letting us try out what we've learned earlier yeah and it feels much more realistic than just sitting behind the desk and listening to someone down there.” (Student 6)*

Students consistently described fieldwork as a central source of motivation and engagement. They reported that this way of learning enhances their commitment to the tasks, fosters hands-on experiences, and encourages active collaboration within student groups. Many students articulated a clear understanding of why they have chosen to study at Svalbard, emphasizing both the academic value of Arctic field experience and the personal significance of being in what they perceive as a unique place.

*“(…) for me is not just about learning things but also just experiencing Svalbard and having a bit of fun (…).” (Student 6)*

*"Yes, yes, you come up here to UNIS to have a learning environment outside the classroom and actually see these things we talk about. But it doesn't feel as much like work when you're out in nature doing measurements. (…) It doesn't feel that intense when we're in the field." (Student 4)*

Some described that this contributes to a sense of community among students:

*“Nobody came to UNIS unwillingly, I think. So in a sense, I think there is, I would say, compared to my home university, there's quite a stronger sense of community for students. And then on top of that, I mean, yes, there are different programs in geophysics, geology, technology, but they all have the focus of the Arctic, and I think everyone who's here loves the Arctic in their own particular way because of the nature, because of... And then that's, I think, kind of like a binding force.” (Student 2)*

*“(…) this going on fieldwork together is really bonding time.” (Teacher 5)*

*"we went on a research or teaching cruise up to kind of the northern part of Spitsbergen for a week and on the way back the ship was in big seas but in the little common room all the students were gathered there, some were reading, some were doing some work. And they were sat, you know, arms around each other in each other's laps. And it reminded me of like a nature documentary where you see a family group of chimpanzees or gorillas (…). But, you know, in a very, you know, kind of bonded family kind of way." (Teacher 2)*

Staff also highlighted the substantial institutional support required to facilitate safe fieldwork, including logistical planning, risk management, and continuous supervision. Before each field excursion, students must be briefed on weather forecasts, avalanche risks, boat safety, and other environmental hazards. Unfavourable conditions can delay or prevent fieldwork, requiring teachers to reorganize course schedules at short notice. In some cases, postponed field activities must be conducted during students' and academic staff free time, creating further logistical and academic pressures.

Indeed, several students mentioned the “Arctic flexibility” as a concept, referring to the frequent changes of schedule and unpredictability in planning.

*“There's this maybe oh no someone is suddenly, I have to leave on Monday so let's squeeze in that last lecture on Sunday evening and just leave with it, this is arctic, what was it called, yeah adaptability, yeah arctic flexibility, yes, so that is also a separate factor here at doing this you have this yeah you can turn crazy at." (Student 6)*

The teachers described that they perceive the students to be less accepting regarding this arctic flexibility than earlier.

*"In one of our last courses, we got lots of complaints from the students because the timetable was changing every day. And in that particular course, the fieldwork was cancelled every single day. The first day the fieldwork was organised for, it never happened on that first day. It was always pushed back, and that was a weather problem. We had a terrible spring. It's just been high winds, gales, and you physically cannot take people out and across the island. (…). And yet the students don't seem to understand that these changes were being enforced on us. We didn't want to do it either. It just makes our life much more complicated. And that understanding that these sorts of things happen doesn't seem to be there as much as it used to be, or maybe they're just not as flexible." (Teacher 1)*

The staff talked about the increase in questions about adaptations, not only for exams, but also in other situations, and for instance more questions related to social anxiety. Some of the needs were regarded more legitimate than others, but overall, staff perceived the students to be more demanding also when it comes to personal preferences. Examples ranged from students who don't want to present in class, who have trouble sleeping in new places, or who don't like the food on the ship.

*“Research it isn't a gourmet restaurant. They provide what they can. They will, of course, make allowances for allergies and so on, but they can't provide you with your particular favourite meal.” (Teacher 1)*

Academic staff stressed the importance of safety training for field-based learning. Students must complete courses on cold-weather clothing, polar bear protection, survival suits, and ocean survival. These activities aim to ensure safety, boost confidence, and enhance learning in Arctic conditions.

*“I'm present and one of our field engineers comes in and we go through exactly where we're going, what we're doing, what the weather is, what the risks are, how to prepare, how to dress for that particular occasion, what food to bring, and I think that is really important for the students as well, as it is for me.” (Teacher 2)*

Students described teamwork in the field as demanding and often intensified by these environmental and logistical pressures.

*“I don't have any special medical issues or something but if you go on a boat for like a week or even more then you have to like prepare for that in a different way than when you just go like one day on field work or something and I think that could also be maybe some more information about that also if you need to share rooms. I think that's also a big topic that there's maybe people that don't want to share rooms with other people but then you go to a small cabin or something and you have to share rooms and toilet situations can be tricky and like all of that stuff.” (Student 6)*

Academic staff reported that medical conditions including severe allergies, chronic illnesses, and mental health concerns can limit students' ability to participate safely, particularly during remote excursions where rapid return to UNIS is not possible. Being on a boat a long way from the civilization makes it more difficult to make adaptations and allergies and other medical conditions may hinder participation and even reduce the learning outcome of the whole group.

*“ (…) the captain refused to do the entire cruise because he was concerned that the students were not going to, that if there was a problem, he had too many students on board that had allergies against this or not that and something else and phobias (…) and so on. And he was too scared to take the students beyond helicopter range and then the weather closed in and helicopter range actually became very short.” (Teacher 1)*

*“But if you have allergies where you go into anaphylactic shock, that's a big problem when you're on a ship. And the ship actually doesn't allow that. (…). But they would carry EpiPens around, all the teachers at least were aware, so that we would know what was our window timeframe in which, okay, if they start showing symptoms, it starts happening, in which to get the EpiPen administered, things like this. And then finally the ship said, okay, they allowed them. (…) But then I went on the ship and they said, OK, we're not going to go to this in this location because it's too far away. And we don't want to take that risk that we can't get this person back to a hospital. So then it affected like actually the entire the plans of the cruise and where we were doing, how we were sampling the locations we went to.” (Teacher 4)*

This illustrates how a present medical condition can influence the risk calculations for the whole group, effectively making one student's health matter a collective pedagogical constraint. Unlike on the mainland, where adaptations typically can be made on individual level, the logistics at Svalbard forces any such accommodation to influence all students in the group. One staff member emphasized that mental health risks, including self-harm and suicidal ideation, are of particular concern in the Arctic context due to isolation and limited emergency access. Especially on cruises:

*“Because on the ship, one thing you have to be very careful about is if anyone has a tendency to self-harm. Because if you... if you have any kind of suicidal thoughts, then you have to actually be locked in your cabin so that you don't do something. (…) Because it's very easy if you want to do something to yourself, you can really do that on a ship. So we have to be very careful about things like that. So both me as the cruise leader and the captain, the bridge, they need to know when there are any of these issues. We haven't got to that or I haven't heard of that.” (Teacher 4)*

#### Ambivalences

A theme in the interviews concerned the paradox inherent in staying at UNIS to study different aspects of the environment while simultaneously contributing to the environmental footprint associated with living at Svalbard. Several participants described this as an ambivalence: they are committed to understanding environmental change, yet their presence inevitably adds to the negative impacts on the natural surroundings. Teachers emphasized that there must be a compelling justification for conducting research and education in Longyearbyen. According to them, students' contributions to environmental monitoring and knowledge production provide such justification. Consequently, student stays are typically short in duration, often limited to a single semester or even less, and fieldwork is prioritized to maximize the scientific value of their presence.

A second form of ambivalence related to students' motivations for coming to Svalbard. While many students are academically driven, the unique natural environment also attracts the so-called “tourist students”. These are students who come primarily to ski while the studies primarily serve as a legitimate reason for staying in Longyearbyen.

*“…some of the courses in winter and spring, especially snow science, avalanche science, there's a lot of people up here who just want to ski.” (Teacher 4)*

The abundance of recreational opportunities, particularly throughout the winter season, often creates distractions that compete with academic commitments according to both students and academic staff. Activities such as skiing, cabin trips, and snowmobile excursions may challenge students' focus on their studies.

*“So I think that balance of trying to... trying to be a good student and also enjoy the nature here is the biggest issue that I see right now.” (Teacher 4)*

### Belonging and identity

The first arena where the students connect with each other is the safety course, a one-week introduction to Svalbard and the threats that one needs to be aware of (e.g., learning to shoot and handle a rifle and knowing how to act if a polar bear appears, clothing and weather conditions, etc.). During this intense week, students often form relationships that will last for their entire stay.

The second arena is the student kitchen. Almost all the students are living in the same student apartment building (Elvesletta), where groups of students are sharing kitchens. Here, they might cook and eat together, play games, or go on trips together.

*“I think they're very loyal to their kitchens as well. Yeah. But I think in terms of belonging at UNIS and at Elvesletta, there's quite a strong sense of belonging.” (Teacher 4)*

*“So I'm very pleased that I share the kitchen. And it's very nice when I get to know them and meet them at school, then I get to say hello to their friends also.” (Student 5)*

The close student community may also pose a challenge related to alcohol, and one student talked quite much about it:

*"But many activities are centred around alcohol and drinking and such. And I think that's a bit awkward. So I feel there is more offers, at least especially in the dark season, I would have liked more activities that do not in a way centre around alcohol. Alcohol is very cheap here at Svalbard.” (Student 4)*

The third main arena is the class. Class size is normally smaller compared to the mainland, seldom larger than 20 students. The teachers tend to be conscious about creating a safe learning environment, using group work and discussions frequently. Much of the field work requires team efforts.

*“And the field work is quite demanding. It requires teamwork, and that builds the group as well.” (Teacher 1)*

*“They're collaborating all the time. They're living together. They're recreating together. They're being educated together.” (Teacher 2)*

So, there are several arenas where the students are almost forced together. At the same time—it is a vulnerable situation because the total group of people is quite small.

*“Or if you feel like you didn't meet or connect with people and you don't really feel like you have someone to do things with, then it's not easy.” (Student 1)*

The relationships with teachers are also closer than usual:

*"It’s nice to be myself. I think it’s really fun with the professor we have out in the field now. We were two days in the field yesterday and the day before yesterday. And then I’ve been sitting right next to her in a boat that takes one and a half hours one way. And just talking about everything and nothing. Just joking about things and talking a bit about subject matter. I think it’s incredibly fun. It’s not as scary to talk to them as it maybe is in other places." (Student 4)*

UNIS does not have specific student clubs or societies, because there is an aim that the students should instead participate in the activities offered for the general population in Longyearbyen. In some of the interviews, especially with staff, we got the impression that many of the students are indeed taking an active role in the Longyearbyen community. One of the teachers told us that they explicitly encouraged the students to take part:

*"If you want to know something about Arctic Safety Management, and if you want to know something about being in the Arctic, then you have to be part of the Arctic. I don't want to hear that you come here and haven't been to Fruen [local pub], or haven't eaten at the Station [restaurant], or haven't been among people. You have to be where the people are. You have to feel it on your body." (Teacher 7)*

However, most staff described the students as in a bubble, forming a UNIS community and organizing their own activities, and if they participate in the more general activities, it is still as a group.

*“I think they mostly stay among themselves. I would say some students are better than me in getting out and doing things in the local community, like, you know, going to volleyball at Svalbardhallen and those kinds of things. But overall, I think it really, they're a little UNIS community and UNIS students. And it's not like they don't participate. Like I said, you know, they go to Svalbardhallen. I see them at like the weekly, we have this quiz night on Mondays at Tio Moncho's. And I do see them as well, but they're like in their group.” (Teacher 4)*

It is, though, another important aspect of the student life in Longyearbyen that they do indeed organize a lot of activities and groups themselves. Most students we talked to said they had a range of opportunities every week, and that it might even be a bit much.

*“I feel like I always have something to do, which makes it hard to do all the things. I just think there are a lot of things that I want to be a part of or that I want to learn about. So I feel like time goes very fast.” (Student 1)*

It seems to foster a certain fear of missing out:

*“But here I'm oversaturated. Like it's too much to happen and it's always a challenge to pick. What you want to do and not be like, oh, well, damn, I'm missing out on doing that other thing, even though I'm doing something fun now, but still being worried about missing out on some other things.” (Student 2)*

The Longyearbyen community is described as difficult to get into, at least by foreign staff.

*“I mean, definitely, I think for people who are not Norwegian, it's very difficult to integrate into the Longyearbyen community. So for those students, I would say that's a big barrier. So they really are restricted to the UNIS community.” (Teacher 4)*

*“And then there are issues in, Longyearbyen is actually highly politicised. And I know that there's, to me it's just water off my back, but for some international members of staff, they feel devalued. because of decisions made in Oslo about access to services for non-Norwegians. I think the community here itself is very supportive and it's very proud of its international identity but there are decisions made elsewhere that directly impact people from an international background far more than they would impact people on the mainland.” (Teacher 2)*

The temporal nature of being a student at Svalbard also seem to contribute to the lack of integration. The locals do not invest much in getting to know people they know will leave shortly. One student said:

*“people sort of can smell your ‘short-termness’ on you, and then they treat you accordingly.” (Student 2)*

This changes dramatically if your status changes, for instance if you go from being a student to having a permanent job. One teacher elaborated on their own experience:

*"(…) I noticed it myself from when I worked here, I remember I had a very specific feeling from the first time I just came and was going to be a holiday substitute. (…). And then when it was nearing the end, the employer asked if I wanted a permanent job, if I wanted to stay. And then I say, yes, I don't have any plans, so what can I say, then, no, I can stay. The feeling, that change in the mood from not only being here for two months, but staying and having a permanent job, was completely palpable. And I have heard others say the same thing, that when you are taken into the warmth of the town among those who live there, you are integrated in a completely unique way. People are nice to each other on a short-term basis, but when you get that embrace of being part of the town on a long-term basis, it's a completely different experience." (Teacher 7)*

The quote suggests that belonging in Longyearbyen is not simply earned through time or effort, but depending on a status change that most students, being temporarily present, can't access. It is not primarily that the students are failing to integrate. Rather, the “short-termness”-smell introduced by Student 2 seems to be a structural feature of the community.

#### Diversity and inclusivity

The students are in most aspects a very diverse group. They come from a range of different countries and cultures, with different background knowledge and study habits and they are used to different levels of expectations.

*“There is no similarity except that they are people. In roughly the same age group." (Teacher 7)*

This contributes to extended learning outcomes, because students learn from each other and see things from different perspectives.

*“So you got to learn a lot from your fellow students as well. I think that was very good.” (Student 3)*

However, the macho attitude and “Arctic heroes” concept came up several times. This points to a stereotypical masculine culture, that may be mirrored in language and behaviour, but also in the lack of universal design and adaptation. A lot of the needed equipment and clothing is designed for men, and it is in general easier for men than women to be out on cruises and field work when it comes to sanitary conditions.

*“(…) if you were to ask Chat GPT to draw you a picture of a polar scientist it would probably be a middle-aged man with a big beard and we're trying to get away from that you know, we're trying to make sure that maybe smaller women can understand that they can play an active role as well you know, that logistically that's always been a bit of a problem because when you start looking at survival suits and so on they tend to always be large sizes.” (Teacher 1)*

Staff at UNIS told us that they are actively trying to combat these stereotypes, making the student environment more inclusive.

*“So you do get harassment, you do get nasty things. And if anyone sees or hears or anything, come and talk to me. If you have no one else to talk to, I'm always here. I try to be available for the students if they have a problem or if they... think I could improve things somehow. So being present is, I guess, one of the important things.” (Teacher 6)*

### Extended roles

Teachers gave many examples of how their professional teaching role at UNIS is extended, both as “*a family*” “*a first responder*” (Teacher 2), “*contact person*”, also during holidays (Teacher 3), “*a therapist*” (Teacher 3) “*a friend*” and “*a mentor*” (Teacher 2), “*counsellors*” or buddy to support students when they are heartbroken (Teacher 3) or a care giver or emergency provider (authors' characteristics of the role) who “*get the EpiPen administered, things like this*”. One teacher gave an example about how they had to support a socially anxious student during a cruise:

*“So then I went to check on her, and she was just completely crying, like, yeah, hysterical. And she just said that it was too much. She has social anxiety to begin with. And just being on the ship in this situation was making it worse, which is completely understandable. So then I was like, okay, guys, just take your time. Spend some time in the cabin. I told her cabin mates to let her be, not to go to the cabin. And we checked on her and everything. And she just needed time. And that was okay.” (Teacher 3)*

Many of the teachers linked their extended role to the harsh learning environment, here exemplified by teacher 3:

*“So the percentage of students that ended up crying in my office kind of took me by surprise. So that was more than one quarter of the whole student body. It's a harsh environment and I try to be present if the students want something and I communicate that I will take their stuff seriously because I know this is a place unlike any other where they've been.”*

The environment was by another teacher described as difficult, particularly for students who struggle:

*I've had students drop out (…) and go back home. and really struggled psychologically up here. So I think that it is a pressure cooker.*

*"(…) People bring their own problems up here, their own background, their own histories, and I think this place can be an amplifier of that. And it is a very difficult environment. There's a lot of emphasis placed on self-reliance up here. And for some, that's amazing. Others, I think that can be something that they struggle with. Especially when perhaps there isn't the provision of services that you would expect on the mainland or in a big city in terms of mental health care." (Teacher 2)*

Teachers reflected upon their limits and lack of competence in dealing with mental health issues:

*"And what I think is really tough is that we also don't have any training in dealing with psychological issues. So as you already mentioned, we have the safety training. And when it comes to things like that, I'm on the ship, if there's any sort of safety issue, I feel very well prepared for dealing with that, and the crew as well. But when it comes to any sort of psychosocial issues, I really, I don't even know how to deal with it. (…) So I think it would be good if we got at least some basic training for this so that we don't make very fundamental mistakes and make it worse in these situation” (Teacher 3)*

Other teachers, also expressed their lack of competence in dealing with “psychological issues” but described that they had to solve them when they appeared during field trips or cruises, and because there was not a present mental health service in Svalbard:

*"(…) I had quite a lot of students come to me with special issues that they have (…). And in terms of these people with issues like social anxiety, then I try to do things like put them in a cabin with... Because you can either have cabins with three people or two people, and most of them are three-people cabins. But I try to do things like, okay, the person who said that they have this, put them in a two-person cabin (…). But it would be really, I would say, on a case-by-case basis right now. And what I think is really tough is that we also don't have any training in dealing with psychological issues." (Teacher 4)*

*"...but they don't have a support system here and that means if there is something that somebody who are struggling (…) or something is happening in their personal life (…) they come to us of course because they don't have the rest of their support system and yeah and that is not something we are professionally skilled to do. (…) sometimes we manage well, sometimes we don't, because it's everything from yeah somebody who is struggling with a partner to somebody whose parent is sick to somebody who thinks about committing suicide so it's the whole range of things. (…) Yeah, where us who are not professionals are not skilled at dealing with it." (Teacher 2)*

The year before the interviews, the student welfare service had external funding for physical presence of a counsellor, and a short-term contract with a psychologist. At the time of the interviews there was no such service in place. Teachers and administrative staff experience an increase in students reaching out to them when they do not have such service physically present, and shared examples about how if affected their work and challenges their roles:

*"I have had those who have come and been "Just want to go home, just want to go home, just want to go home", then it is a matter of talking to them a little and listening, what is it about. But none of us are professionals, but we have had Samskipnaden in Tromsø [the student welfare centre, authors’ comment]. So I've called there and gotten good advice there, and asked the students to get in touch as well, but I would argue that having an advisor here physically, i.e., when we look at what they've done since they started (...) it's, yes, we have to have someone, and someone who actually has it as a field of expertise." (Administrative staff)*

Staff reported uncertainty about whose responsibility it is to follow up students who struggle, as there is no mental health system or psychologist to refer the student or themselves to in difficult situations:

*“So in terms of who is responsible, that's a difficult question to answer. It's unclear. (…) I think a lot of us feel that we would be on the front line dealing with these things and not really sure where to take it or how to access those services (…) either to direct students to that or for our own well-being when dealing with difficult situations.” (Teacher 2)*

Students gave many examples of how they trust their teachers and could ask them for help in challenging times:

*"Yes, I actually think that the professors were very... You could easily talk to them, at least. That was no problem.*

*Interviewer: So if there were to be any problems, you would go to the professors first?*

*Student: Yes, I actually think so. And asked where I could have turned, yes." (Student 3)*

Nevertheless, there are several examples that the teachers have had students who struggled, were depressed, anxious or homesick. One of the teachers believed that students might have the perception of mental health issues not being accepted, and that this topic should be addressed more openly:

*"I don't think it's discussed enough. (…) I do make reference not to specific cases, but probably say there have been cases of students attempting suicide and... You know, they have people that have been asked to leave the island for their own benefit. And there are rumours that are counterproductive amongst the students, that if you were to report or demonstrate mental health problems, you're sent away off the island. And I think they really want to be here. So I think despite what services may be available to them, I think a lot of them are very reluctant to actually address those problems because they're fearful that they'll be asked to leave Svalbard." (Teacher 2)*

Another teacher pointed at the safety course as a possible arena to talk about mental health:

*"So it's not very much addressed, I would say. Yes, the safety course is primarily dealing with physical safety. And that I think they do really well. But in terms of mental health, I would say that we are quite poor in terms of preparing students and also having resources." (Teacher 4)*

Students and teachers shared examples about how they become affected when they experience that students are having serious mental health issues, and call for better routines:

*"So there was a definite need for one better handling, but for a real debrief that my colleague in my department actually did give them. But that wasn't an ideal situation. (..) the kind of systemic level would be useful." (Teacher 2)*

The administrative staff are also given extended professional roles and responsibilities, and reflected upon that:

*“I don't have that expertise, yes, I have experience, but there are a lot of strange things you come across. Everything from parents who call, and ask for, that is, have not heard from, from, yes, from the child, and are worried, and then it turns out when you investigate a little, well, we can't give any information, it's a question of a person who is over 18, and all that, and when they don't want to have contact with the parents, because there is something far behind, there is not much we can do.” (Administrative staff)*

Students are dropping by the office of administrative staff both when they have « *practical questions*” and when they “*have a bad day*” (Administrative staff). Moreover, the discussions in the focus group interviews showed that UNIS employees are very “*service minded*”. One of the teachers gave an example of how they discussed complex cases with the administrative staff or even referred a student to a hospital:

*"…normally, if small things, they come only to us, or it can also be more serious things, of course, but serious things we would never handle as a professor head of department alone we would take it to the study administration first they would weigh in sometimes they have taken it on sometimes some has been involved sometimes hospital has been involved so it depends on what it is." (Teacher 3)*

Students, administrative staff and teachers all described close relationships between students and teachers, using phrases or metaphors like “*open door policy*” (Administrative staff), “*camaraderie and group identity with your students*” (Teacher 1) and “*a little family*” (Teacher 2). One of the teachers underscored how these close relationships between teachers and students was positive for their learning process:

*"There are some special things with coming here and being part of a group, being in small classes, being close to the teachers, that are very positive for most students learning.” (Teacher 3)*

Both students, teachers and administrative staff expressed a need for physical presence of a mental health service or a clinical psychologist, for the whole town, as well as for the students:

*“There is a push from many people in town, many organizations in town for having a psychologist here. I think that would be very good for UNIS. (…) So the local community, and UNIS students, all, they see a need for better support systems.” (Teacher 3)*

*“We don't have actually psychological help in Longyearbyen and this is a problem for the entire society at Longyearbyen not just for the students I think.” (Teacher 4)*

The only alternative is an online service, but both students and staff express that this is not enough and cannot replace physical presence of a therapist:

*"Like, I would have to actually find a psychologist on the mainland somewhere. It's not just like, okay, you call this person right away. And I have enough Zoom fatigue that I don't want to do more meetings online. So, yeah. So instead, I'm like, just get through it. Do the bare minimum that I need to for my own health. But, yeah, I think this is a big problem in Longyearbyen in general." (Teacher 4)*

*"I actually had one day where I was... I don't know, feeling... stuck in that way or I had a few times, but one day where it was bothering me more, yeah, and then I didn't really know what*

*I: so what would you have needed, you think, in that situation?*

*S: I mean i was looking up uh like therapy session or something but it seemed a bit, quite a lot of hassle to get a hold of something somewhere. I know there is like you can book an online talk session*

*I: Do you think it would be different if she was actually here? Or if there was a person located here?*

*S: Probably, yeah. Like, then it's less effort to go and talk to someone" (Student 1)*

*"(…) there used to be a student counsellor here I guess she was aware of and whatever funding for her presence up here ran out or something and so now she's not here anymore but this I think was quite a relevant offer and I think it's very sad that that's not there anymore. We had a guest from student parliament two weeks back here who was also interested in that topic and there with talks with several people from student councils, it's apparent that several people have used the digital offering that is there now, but would definitely 100% have preferred to talk to someone in person. Because it's a much lower barrier going to somebody who's sitting in an office here than looking up how to get an appointment. Making an appointment, logging in for the appointment, remembering the appointment.” (Student 2)*

## Discussion

We interviewed students and teachers at UNIS, the university centre in the northernmost town in the world, where nature with all its joys, challenges, and dangers is always at the doorstep. Being a student at UNIS means studying and doing fieldwork under the midnight sun and polar nights and involves being able to handle a rifle to protect yourself and your peers from polar bears. Being employed there means having responsibilities way beyond what a conventional teacher or study adviser role include. The setting seems dramatic and may fuel worries and difficulties, but it is also an opportunity to getting close to nature and people around you. The extremity in the situation nevertheless makes UNIS a critical case to study.

Our aim was to explore how students, teachers and administrative staff at UNIS perceive student's well-being and learning in this specific educational setting and how these are related. Using the Whole university approach (Hughes & Spanner, 2024) was helpful to identify how the different aspects of the student life at UNIS are connected to each other. It helped us see a clear picture of how the strong educational qualities at UNIS, especially field-based learning, also creates vulnerabilities across the four different domains of Learn, Support, Work, and Live. We will discuss our main results while connecting them to the different domains of the framework. While Support is the second domain in the original presentation of the framework, we will address it last here, because the discussion of this domain largely builds on the discussion of the three others.

### Learning in the Arctic

The Learn domain in the framework emphasizes inclusive and flexible pedagogy, support for learning and engagement, and academic and social integration (Hughes & Spanner, 2024). At UNIS, learning is very much about nature, in nature. The main reason students are there, is because this is where the field work can be conducted. The large amount of field work and cruises is undoubtedly a valuable strength for the learning process. This type of active experience-based learning promotes deep understanding and engagement (Kolb, 1984) and includes students into the scientific community (Lave & Wenger, 1991). These shared experiences in demanding environmental conditions appear to promote collaboration and commitment, described as a “binding force”, strengthening both academic and social integration.

However, being dependent on nature also makes the learning environment vulnerable to external conditions, primarily related to weather, which might cause sudden changes to the schedule. Students and staff need to be prepared for this, to be flexible and able to adapt. In addition, it is more difficult to access necessary medication and other treatments, and especially on cruises, this creates challenges. A systematic review on disability and fieldwork in natural sciences points to how reduced planning, flexible alternatives, and support may reduce the learning outcome of students with different challenges (Chasen et al., 2025). The learning outcome for a whole group of students may in fact be reduced when a ship captain doesn't want to take risks going too far from land or helicopter range when students with serious allergies or suicide thoughts are present onboard. Moreover, the mandatory requirement to carry a rifle outside Longyearbyen creates a barrier, as students without access to one become reliant on fellow students to conduct required field work.

The close relationships between students and staff most likely contribute to the strong academic integration and identity, and to extending the arenas for learning opportunities beyond the class schedule. This closeness also makes it possible to be more flexible and adaptive in relation to the individual student.

Most students share a love for nature, which creates several ambivalences. One is connected to being part of the problem, i.e., investigating climate change or specific Svalbard conditions while being responsible for their own environmental footprint on the island. Another ambivalence is connected to the balance between spending time in nature doing leisure activities and doing the study work needed to learn and to pass the courses.

### Beyond work

The Work domain concerns staff well-being and development. This includes ensuring the necessary competence to support students' well-being, while also maintaining healthy boundaries (Hughes & Spanner, 2024). Staff at UNIS occupy highly extended roles. Such extended roles are not unique in higher education and has been noted elsewhere (e.g., Bennett et al., 2024; Martinez et al., 2024). However, the characteristics specific to UNIS, like geographical isolation, small class sizes, frequent fieldwork and close students-staff relationships, combined with the limited mental health services, are likely to amplify the extended responsibilities.

Strong relationships between staff, both academic and administrative, and students have clear benefits. Strong trust supports learning and sense of belonging, and the closeness makes it easier to identify challenges, make necessary adjustments and meet individual needs. However, these ties are also vulnerable and person-dependent and place a quite large responsibility on staff. This responsibility is often implicitly expected, rather than explicitly defined, which blurs boundaries, and may complicate issues related to privacy and information safety. Staff report that, despite wanting to help, they often lack the training to support students' mental health effectively. This creates a dilemma: providing training would better equip staff but could also formalize caregiving as an expected part of the teacher role. Not all teachers may want that extra responsibility to be made explicit (Brewster et al., 2022). On the other hand, for many of the UNIS staff, caregiving is already part of the job, and insufficient competence can undermine their well-being. Deliberate training and clearer role definitions could increase predictability and strengthen staff's confidence in handling such situations. Educated therapists are often trained in setting and maintaining such boundaries, while higher education teachers and administrative staff typically are not and may need support to not overextending themselves. The extra workload associated with extended care responsibilities should be acknowledged and matched with appropriate resources provided to safeguard staff's well-being and coping (Brewster et al., 2022).

### Living in Longyearbyen

The Live domain covers residential accommodation, social integration and belonging, mentally healthy environments, supportive communities, and physical learning environments (Hughes & Spanner, 2024). Our data indicate that they thrive and enjoy their stay, also outside studying. It is evident in all the interviews with both students and staff that the students at UNIS develop a strong identity and sense of belonging to UNIS. Dropout rates are very low, and often students want to come back after they finished their courses, to do more courses or write a thesis. What is less clear, is how this looks when it comes to Longyearbyen and the community outside of UNIS. The Norwegian policy in Svalbard strengthening the connection to the mainland, may contribute to a perceived social hierarchy in Longyearbyen. Especially foreign staff mentioned this, while the Norwegian staff did not perceive it in the same way. However, most participants, regardless of nationality and status, describe that Longyearbyen may be difficult to get into when you are only temporarily there. At the same time, the fact that the students share this temporality might also contribute to the strong sense of community among them.

The typical UNIS students share a love for nature and will spend time there outside of studying. However, the threat posed by polar bears is significant. Their presence on the island is one of the things that makes Svalbard unique, but it also hinders students traveling freely on the island. Without access to a rifle—or to someone equipped with one—it is not possible to go outside the settlement in Longyearbyen, hence leisure activities are more limited.

### A need for support

The Support domain involves all kinds of resources the student population would need during their education. A Whole university approach combines well-resourced, accessible mental health services with proactive efforts to help staff and students develop the skills and understanding required to sustain their well-being (Hughes & Spanner, 2024). At UNIS, mental-health support service is in large lacking. The small community, weather conditions, and isolation may indeed amplify problems already present, for instance depressive thoughts and anxiety, making such a service even more important. Students generally trust their teachers with their challenges and problems, but teachers report they do not feel well-equipped to handle everything students bring.

There are several strengths to teachers holding a care-role. First, support is accessible through someone the student already knows and understands how to approach. This is especially beneficial for vulnerable students who find it difficult to ask for help—they can just stay with their first point of contact instead (Bennett et al., 2024). Second, combining teaching and care responsibilities can make it easier and faster to arrange and implement adjustments: teaching and administrative staff can identify issues and often put solutions into practice directly, without routing requests through third parties or having to exchange sensitive information.

However, an on‑site mental health service is essential in this context. Teachers and advisers are neither trained nor responsible for providing clinical assessment, diagnosis, risk management, or ongoing psychotherapy—core functions that require trained mental health professionals. In an isolated setting such as Longyearbyen, the capacity for immediate, face‑to‑face assessment and intervention is particularly important, both for acute crises, therapy, and for implementing mental health prevention and promotion initiatives.

The need for such service is reinforced by the rise of student mental health problems, which also carry academic consequences. Psychosocial factors such as belonging, social support and self‑efficacy are associated with both grades and persistence (Swanson et al., 2021), and an increasing prevalence of mental health difficulties in student populations, thus warranting institutional responses (Storrie et al., 2010). Importantly, research on student perspectives emphasizes that acceptability and uptake of services improve when support is visible, low‑threshold and embedded within campus life rather than solely remote or ad hoc (Priestley et al., 2022). In the UNIS context—where isolation, seasonal stressors and intensive fieldwork amplify risk—these findings argue strongly for a co‑located, on‑site clinical resource integrated into teaching and residential routines (complemented by e-health as needed), both to protect health and to safeguard learning outcomes.

The special environment at Svalbard promotes engagement, learning and social integration, but exposes the students to weather dependent breaks and postponements, logistic barriers connected to access to guns, and medical and psychological risks at cruises and field work. These challenges from the Learn domain cross into the Work domain because teachers often enter informal caring roles without formal training, and into the Live domain because the social life both promotes sense of belonging and a strong identity but may also amplify social exclusion for some students. Furthermore, the teachers' professional roles may be challenged when they have to act as “family”, “therapist”, “first-responder” or “counsellor”. Finally, the findings in the Support domain emphasize the need for local mental health services, training for staff, and more clearly defined roles, boundaries, and routines.

### Implications for policy and practice

Based on our findings and the Whole university approach, we present some recommendations that UNIS and similar educational institutions could consider. The special learning environments and educational contexts could be considered integrated in local academic development programs to prepare teachers better for the extended role. Following this, it is important to ensure that students are also in fact prepared, not only for the physical environment, but also for how to deal with potential mental health issues, and for the flexibility needed to achieve the learning outcomes. To safeguard both academic quality and equitable participation, field courses ideally should include structured information, individual risk assessments, alternative learning activities, and procedures for rapid access to medical and psychosocial support (Chasen et al., 2025). Using principles for universal design for learning helps to preserve field experiences as a pedagogical strength while making them accessible to a diverse student body (Vander Kloet & Lien, 2026). Secondly, we would recommend integrating mental health topics in the preparation of students, for instance during the safety course, and we would recommend addressing both student and staff mental health and well-being in professional development initiatives for staff. This could include developing guidelines for dealing with mental health issues, as well as discussing role definitions, boundary-setting, and institutional recognition of the additional workload. Thirdly, we strongly recommend ensuring access to professional mental health services in Longyearbyen. Finally, we encourage staff and leadership at UNIS to use our findings as a guide for developing tools, protocols, and training structures, and to ensure that the close cooperation between staff, students, and the welfare service are further developed.

This study has some limitations, primarily connected to the recruitment of participants. The student participants were recruited through posters and announcements through different channels. Even though we believe most eligible students had a real opportunity to choose to participate, self-selection is a potential bias. For the teacher group, we recruited strategically via existing networks and contacts, and many of our final participants had previously been, or were currently, involved in one of two centres for excellent teaching. Hence, they may be more engaged in student learning environments than the general teacher population. The administrative staff sample comprised three participants in one focus group. However, since the total number of eligible staff were quite small, we believe that our participants have reflected the whole groups well.

## Conclusion

Our results show that students at Svalbard are highly motivated and engaged. They experience a pedagogical environment of high quality and a psycho-social environment that make them learn well and feel that they belong at UNIS. Teachers are committed to their work, they are pedagogically trained, and they have a culture of discussing teaching and learning together. Yet, our results also show that there is a clear need for psychological support at UNIS (as well as in Longyearbyen). The context in Longyearbyen may amplify challenges, and even though most students cope well, the ones who doesn't, have few places to go for professional help. In many cases, they approach teachers, who often have to take more responsibility for student well-being than the teacher role normally comprises and what they are trained in. Addressing this calls for a Whole university approach integrating mental health promotion across every part of the study life (Hughes & Spanner, 2024), with strong interdisciplinary collaboration between teachers, advisers, student welfare centre counsellors, and the students themselves. While expanding mental health services in Longyearbyen and at UNIS is essential, faculty and administrative staff development should also include training on student mental health, boundary-setting, role definitions, and self-care to ensure faculty and administrative staff are prepared and supported.

## Data Availability

The participants of this study did not give written consent for their data to be shared publicly, so due to the sensitive nature of the research and the fact that the data may contain potentially identifiable information, supporting data is not available.
